# Supplementary material for: Characterisation of Bacteriophage vB_SmaM_Ps15 Infective to Stenotrophomonas maltophilia Clinical Ocular Isolates
Source: Viruses. 2022 Mar 29;14(4):709. doi: 10.3390/v14040709 (PMC9025141; doi:10.3390/v14040709)
Supplement: Supplementary file 1 [file viruses-14-00709-s001.zip › SUPPLEMENTARY 1 - new.pdf]

SUPPLEMENTARY 1.

Stenotromohomonas maltophilia strain fingerprinting

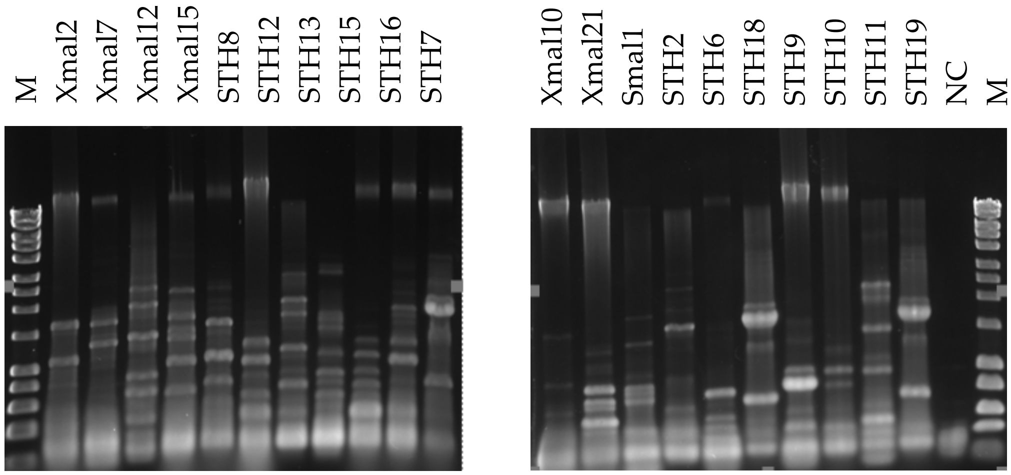

Figure S1. Repetitive-PCR fingerprinting of *S. maltophilia* ocular isolates. Single colonies were used as templates and amplified using a single primer BOXA1R. The PCR products were run in 1.0% (w/v) agarose gels in 0.5x TBE buffer (Tris-borate-EDTA, pH 8.0) at a constant 80 V for 75 min. M - Molecular size marker HyperLadder™ 1 kb. NC - Negative control. The profiles were visually compared.

Transmission electron microscopy of the phage vB_SmaM_Ps15

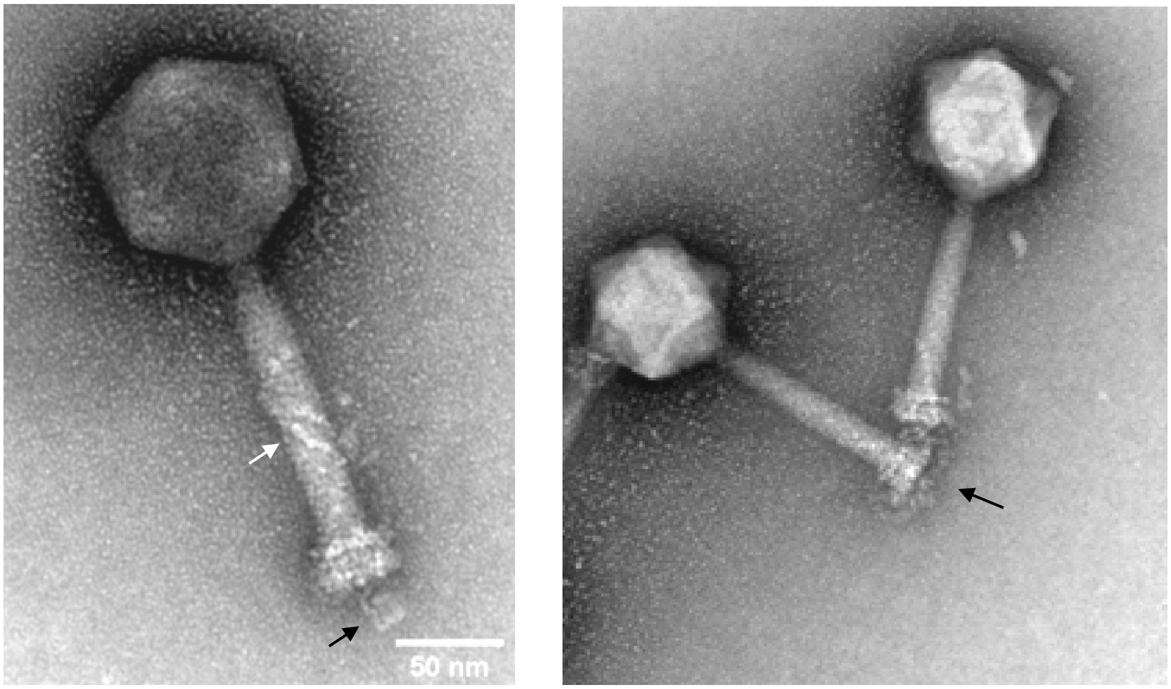

(a) (b)

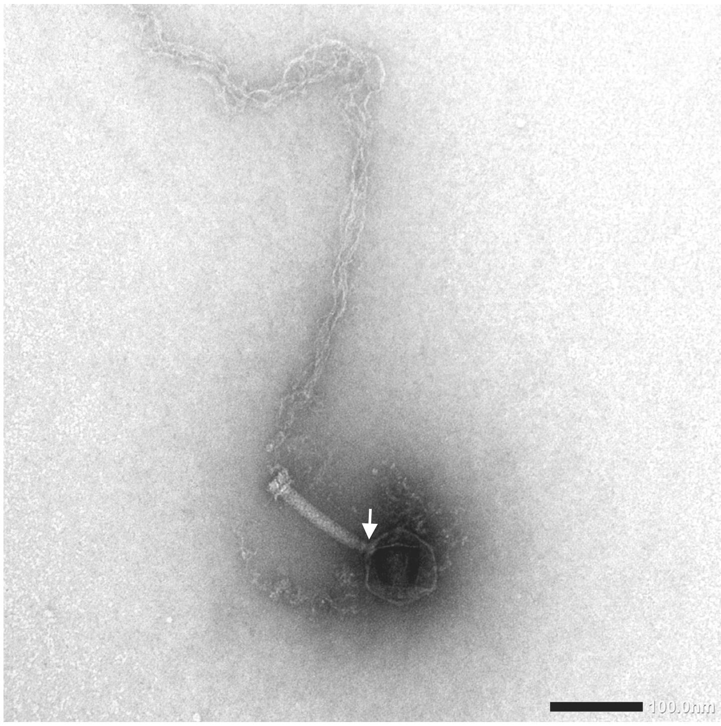

(c)

Figure S2. Transmission electron micrographs of vB_SmaM_Ps15 phage particles. a) An intact phage particle shows surface capsomers of a hexagonal capsid with perfectly straight sides. Short tail fibers are below the rim of the baseplate with folded tail spikes (black arrow). Proteinous extensions are wrapped around the tail (white arrow). b) The image shows an internal content of phage heads. The hydrophobic portion of the DNA (bases) does not bind metal stain and produces light areas. The tail fibers and spikes are in an extended conformation (black arrow). c) The head of the virion has an electron lucent hexagonal outline and appears to be empty. The filaments, which are likely DNA threads, suggest a release of viral genome from the head, which is emptied. The portal structure (valve) is at the proximal end of the tail core (white arrow) and well-differentiated, ordered short tail fibers are visible. Negative staining with 2% (w/v) uranyl acetate (UA). Magnification x 60 K.

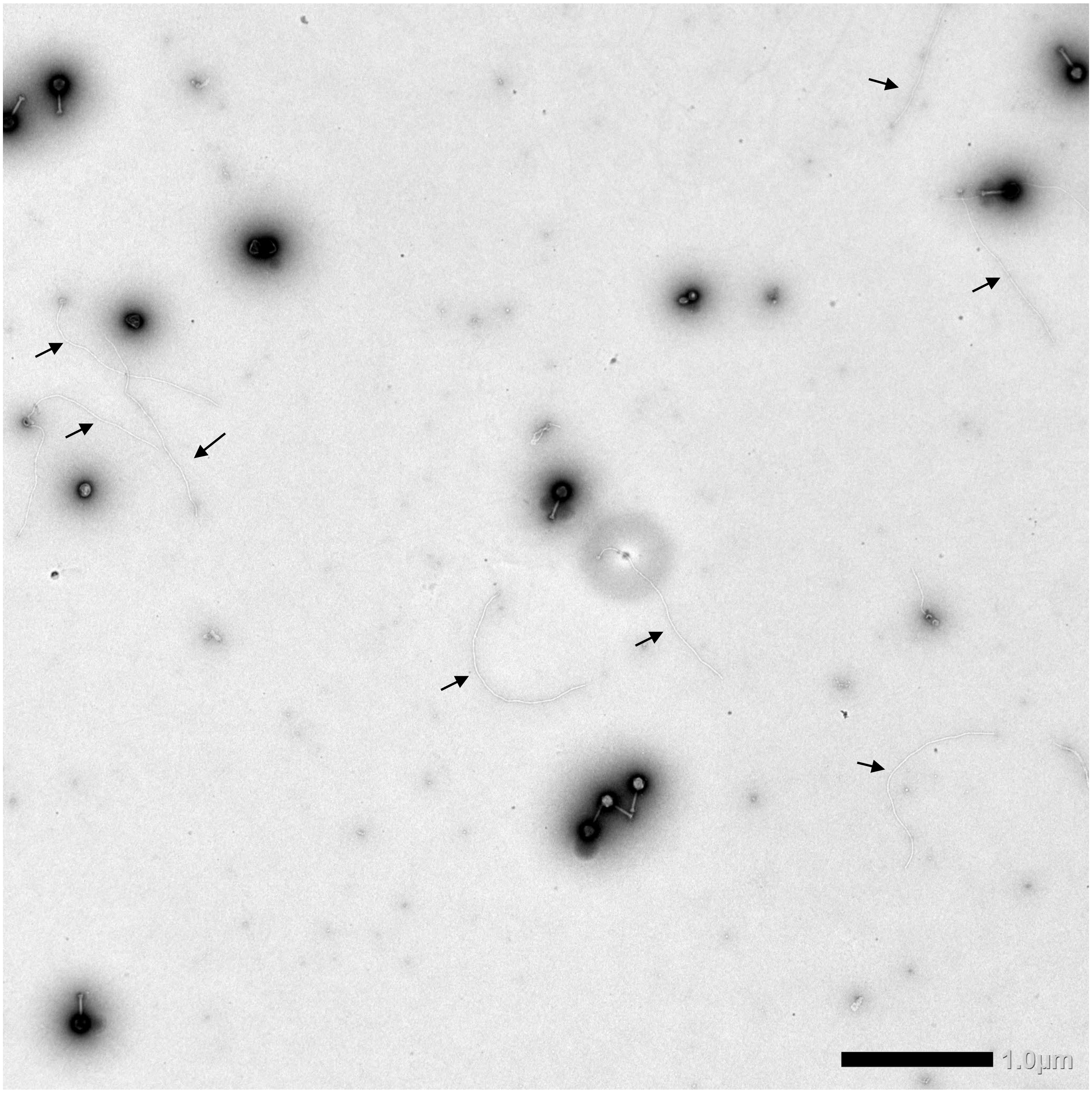

Figure S3. The electron microscopic field showing the content of the original Ps15 phage lysate. Black arrows point to the filamentous structures present in addition to the *Myoviridae* type particles. Negative staining with 2% (w/v) uranyl acetate. Magnification x15 K.

GC and AT skew plots of the phage vB_SmaM_Ps15 genome

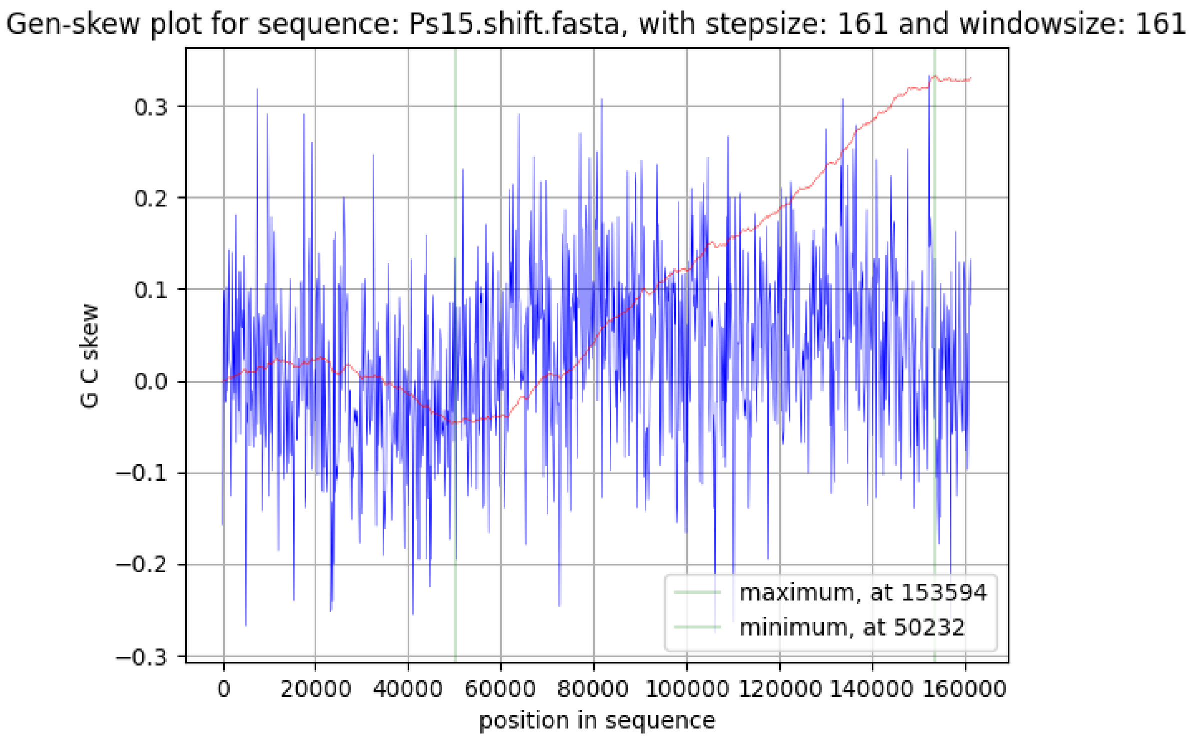

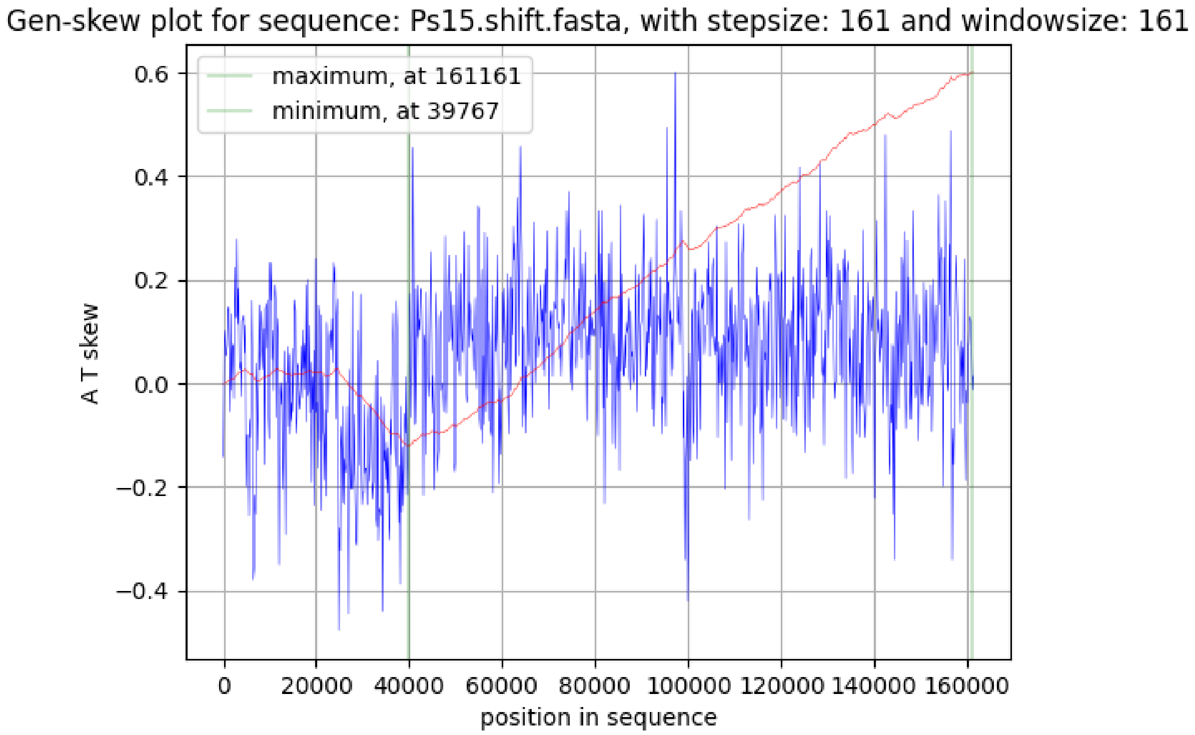

Figure S4. GC skew (top) and AT skew (bottom) plots of vB_SmaM_Ps15 whole genome.
